# Improved Long-Term Survival with Edaravone Therapy in Patients with Amyotrophic Lateral Sclerosis: A Retrospective Single-Center Study in Japan

**DOI:** 10.3390/ph14080705

**Published:** 2021-07-21

**Authors:** Hideki Houzen, Takahiro Kano, Kazuhiro Horiuchi, Masahiro Wakita, Azusa Nagai, Ichiro Yabe

**Affiliations:** 1 Department of Neurology, Obihiro Kosei Hospital, Obihiro 080-0024, Hokkaido, Japan; tk76@hotmail.co.jp (T.K.); horiuchi-kazuhiro@hotmail.co.jp (K.H.); masawkt-hok@umin.ac.jp (M.W.); leukippus0116@gmail.com (A.N.); 2 Department of Neurology, Hakodate Municipal Hospital, Hakodate 041-8680, Hokkaido, Japan; 3 Department of Neurology, Faculty of Medicine and Graduate School of Medicine, Hokkaido University, Sapporo 060-8638, Hokkaido, Japan; yabe@med.hokudai.ac.jp

**Keywords:** amyotrophic lateral sclerosis, edaravone, survival rate

## Abstract

Reports on the long-term survival effect of edaravone, which was approved for the treatment of amyotrophic lateral sclerosis (ALS) in 2015 in Japan, are rare. Herein, we report our retrospective analysis of 45 consecutive patients with ALS who initially visited our hospital between 2013 and 2018. Of these, 22 patients were treated with edaravone for an average duration of 26.6 (range, 2–64) months, whereas the remaining patients were not treated with edaravone and comprised the control group. There were no differences in baseline demographics between the two groups. The primary endpoint was tracheostomy positive-pressure ventilation (TPPV) or death, and the follow-up period ended in December 2020. The survival rate was significantly better in the edaravone group than in the control group based on the Kaplan–Meier analysis, which revealed that the median survival durations were 49 (9–88) and 25 (8–41) months in the edaravone and control groups, respectively (*p* = 0.001, log-rank test). There were no serious edaravone-associated adverse effects during the study period. Overall, the findings of this single-center retrospective study suggest that edaravone might prolong survival in patients with ALS.

## 1. Introduction

Amyotrophic lateral sclerosis (ALS) is a severe neurodegenerative disease characterized by gradual loss of upper and lower motor neurons. The natural course of ALS includes progressive muscle weakness and atrophy of all four extremities, bulbar palsy, and death due to respiratory failure within a few years [1]. Riluzole, the currently used therapeutic for ALS, has limited efficacy.

Edaravone is a free radical-scavenging drug that eliminates lipid peroxides and hydroxyl radicals that damage neurons [2]. Edaravone was initially shown to improve the modified Rankin scale score 3 months after cerebral infarction in patients who were treated within 72 h of disease onset compared to the placebo group [3]. Therefore, since 2003 edaravone has been commonly used in Japan as a therapeutic agent during the acute phase of cerebral infarction. In addition, Japan was among the first countries to approve edaravone for the treatment of ALS in 2015. Subsequently, the Food and Drug Administration approved edaravone for the treatment of ALS in 2017 [4]. A placebo-controlled phase III trial of edaravone in patients with mild ALS demonstrated that, compared with placebo, edaravone led to a significantly smaller decline in revised ALS functional rating scale (ALSFRS-R) scores over 24 weeks [5]. A follow-up report of the trial further revealed that edaravone was beneficial through 48 weeks [6]. However, only one study [7] has reported the effect of edaravone on survival of patients with ALS. Although that study showed survival rates were significantly improved in patients treated with edaravone compared to those not treated with edaravone, the average treatment duration was 8.8 months, which was relatively short. Therefore, in this retrospective study, we aimed to determine long-term survival rates in patients with ALS treated with edaravone.

## 2. Results

The cohort of 45 consecutive patients with ALS comprised 22 patients, including 14 male and 8 female patients, who were treated with edaravone for an average of 26.6 (range, 2–64) months, and 23 patients, including 14 male and 9 female patients, who were not treated with edaravone. The clinical characteristics of the study cohort are presented in Table 1. Because distributions of all continuous variables in both groups were analyzed for normality using the Shapiro–Wilk test (*p* > 0.05), Student’s *t* test was performed to compare the variables in both groups. Although the mean age at disease onset tended to be lower in patients treated with edaravone (edaravone group) than in those not treated with edaravone (control group) (64.8 ± 11.0 vs. 71.4 ± 12.5 years), the difference was not statistically significant. Fourteen patients (60.9%) in the control group took riluzole for ALS treatment, but no significant difference was found in the frequency of use between the control and edaravone groups. Additionally, there were no differences in initial symptoms, average duration from onset to first visit, average body mass index (BMI), and disease severity based on the Japanese classification between the two groups. Furthermore, there were no differences in other clinical characteristics, percutaneous endoscopic gastrostomy, and noninvasive positive-pressure ventilation between the two groups.

**Figure 1:**
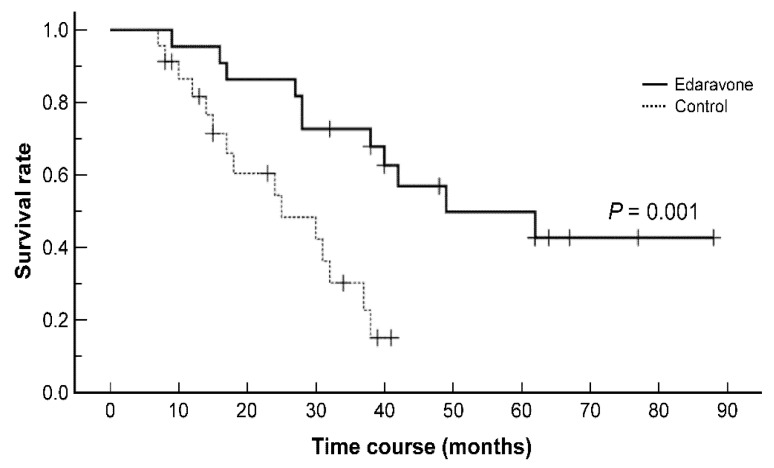
Kaplan–Meier survival curves of patients with amyotrophic lateral sclerosis showing a significant difference in the survival rate between the edaravone and control groups (*p* = 0.001, log-rank test).

Figure 1 shows the Kaplan–Meier survival curves of the edaravone and control groups, revealing that the survival rate was significantly improved in the edaravone group compared to the control group (*p* = 0.001). The median survival durations were 49 (9–88) and 25 (8–41) months in the edaravone and control groups, respectively. Two patients in the edaravone group underwent tracheostomy positive-pressure ventilation (TPPV) at 16 and 62 months following the onset of disease.

The results of the multivariate Cox proportional hazard analysis are shown in Table 2. The hazard ratio to survival of edaravone adjusted by age at onset, treatment with riluzole, and disease severity grade on baseline remained statistically significant (adjusted hazard ratio = 0.36, *p* < 0.05, 95% CI 0.14–0.98).

The reasons for discontinuation of edaravone treatment were worsening of ALS in seven cases and patient request in two cases. The discontinuation of edaravone due to adverse effects was not necessary in any of the cases in the present study. One patient experienced eczema in the palms during edaravone administration, which was easily tolerated with topical steroid ointment. This patient thus continued to receive edaravone and was included in the analysis.

## 3. Discussion

Edaravone is a free radical scavenger that reduces oxidative stress, which plays a role in the disease progression and exerts a cytoprotective effect against degeneration of neuronal cells [8]. Therefore, the lack of protection against neurologic damage leads to the death of motor neurons [9]. These mechanisms demonstrate that edaravone is effective in delaying the progression of symptoms in animal models of ALS [2] indicating its clinical application. The approval of edaravone for marketing and manufacturing in Japan in 2015 was followed by its approval for the treatment of ALS in the USA by the Food and Drug Administration in 2017 [4]. A phase III randomized double-blind study was performed to determine the safety and efficacy of 60 mg/day edaravone administered intravenously for 2 weeks each month in patients with early-stage ALS [5]. The trial results showed a reduction in the ALSFRS-R score in favor of edaravone, compared to the placebo group. A phase III open-label clinical trial examined the long-term efficacy of edaravone in patients with ALS [6]. The trial comprised 123 patients, including 65 patients treated with edaravone for 48 weeks and 58 patients treated with placebo for 24 weeks before switching to edaravone for another 24 weeks. The study results indicated that edaravone was beneficial even after the 6-month placebo treatment and that edaravone was able to maintain its efficacy for 1 year. However, one major limitation of these studies was the presence of several assumptions made regarding the decline in ALSFRS-S scores. The purpose of these studies was to examine the results of edaravone treatment within 1 year for patients in the early stages of ALS.

We conducted the present retrospective study to address the limitations of previous studies and to assess the effect of long-term of edaravone treatment on survival in patients with ALS. Our analyses revealed the long-term beneficial effect of edaravone in ALS for up to 64 months, with prolonged survival of the patients treated with edaravone compared to those not treated with edaravone.

In the study institution, the treatment approach for ALS has not significantly changed since 2013. The only update, which was introduced after 2015, is the addition of edaravone treatment as an option for patients who wish to receive it. In the current study, the patients who chose edaravone treatment were enthusiastic about everything related to survival, which might have contributed to the observed prolonged survival. In addition, the edaravone group also included two cases of flail-arm type in which survival time is generally said to be as long as that in other ALS types [10]. The rates of ALS types were not significantly different between the edaravone and control groups. Although a comparison of changes in the ALSFRS-R score between the two groups would be desirable, similarly to that conducted in other studies, this parameter was not determined in all 45 patients in the current study, which was based on real-world data. Therefore, we did not include the ALSFRS-R score in our analyses. Furthermore, the shortest treatment period was 2 months in the edaravone group, although the average dosing period was 26.6 months. However, the patients with very short treatment durations were not excluded from our analyses, as the goal of the present study was to evaluate real-world data.

In patients with ALS, a low BMI at first visit is a poor prognosis factor [11], whereas nutritional management by PEG has been reported as a good prognostic factor [12]. There were no statistically significant differences in the rate of PEG after diagnosis and the BMI at first visit between the two groups; therefore, the possibility that nutritional management might have affected the survival effect of edaravone was minimal in the present study. The edaravone group tended to be younger with a milder severity grade than the control group, although this was not statistically significant (Table 1). A high proportion of treatment with riluzole in both groups may also have modified the effects of edaravone. Multivariate Cox proportional hazard analysis performed for the purpose of adjusting the interactions of these factors still confirmed the survival-prolonging effect of edaravone.

Previous phase III trials demonstrated that the decline in ALSFRS-R score in a 48-week period was smaller in the edaravone group than in the placebo group [5,6]. However, other retrospective studies from Italy reported that edaravone provided no benefit in comparison to the control group over 6 months [13] and 12 months [14]. These contradictory findings might be due to the use of historical medical records for the control groups. Furthermore, in both studies, the edaravone-treated patients were significantly older than the control patients, although there was no significant difference in the ALSFRS-R score at treatment initiation between the two groups in both studies. The mean ages of the edaravone and control groups were 65.0 and 60.5 years, respectively, in the study by Fortuna et al. [13] and 60.0 and 55 years, respectively, in the study by Lunetta et al. [14]. The difference in the ages of patients between the two treatment groups might explain the lack of a beneficial effect from edaravone. The age of disease onset is a prognostic factor that strongly affects survival duration in ALS [15]. In the present study, although the mean age was higher in the control group than in the edaravone group, the difference was not statistically significant; therefore, age was not a confounding factor in the analyses.

Comparison of survival with a control group is an easy approach to determine the long-term effects of edaravone. One clinical trial evaluating riluzole for ALS demonstrated not only a significant change in the functional score but also a significantly higher survival rate with riluzole compared to control [16]. A study by Okada et al., which had a similar aim to that of the present study, showed that the median survival duration of patients with ALS treated with edaravone was 61 months, which was longer than that of patients not treated with edaravone (32.5 months) [7]. However, the average treatment duration of that study was shorter than that of the present study (8.8 vs. 26.6 months). Additionally, in the study by Okada et al. [7], the average ages of the edaravone and control groups were 62.0 and 67.2 years, respectively, and, albeit not significantly different, the control group tended to be older, similar to that observed in the present study.

This was a retrospective single-center study with a relatively small number of patients, which was a major limitation. The small number of patients included in the present study also hindered our ability to perform subgroup analyses due to the lack of statistical power. Future studies are warranted to increase the number of enrolled patients and to compare the survival effects of edaravone based on age and site of onset. A currently ongoing post-market study in Japan is following the disease course of 700 patients with ALS over a 5-year period [17]. Although the study is not placebo-controlled, the enrollment of a large number of patients with ALS will enable subgroup analyses and comparison with the reported natural course of disease.

This is the first study to demonstrate that long-term treatment with edaravone is not only beneficial but also safe. Several serious adverse events, including acute kidney failure, were reported soon after the introduction of edaravone for the treatment of acute stroke in 2001 [18], whereas no serious adverse events occurred as edaravone was not administered in patients with renal dysfunction as part of the standard protocol [5,18]. Previous studies have reported that serious adverse events associated with edaravone treatment are not common in patients with ALS [19,20,21]. Consistent with that finding, the adverse events of long-term edaravone treatment were minor and easily tolerated in the present study.

## 4. Materials and Methods

### 4.1. Study Design

Forty-five consecutive patients with clinically definite or probable ALS who were diagnosed using the revised El Escorial criteria [22] and treated at Obihiro Kosei Hospital between 2013 and 2018 were included in the study. The patients were categorized into those treated with edaravone and those who were not treated with edaravone (controls). Edaravone became available for patients with ALS in 2015, and its use was based on patient preference and the absence of renal dysfunction according to the standard protocol, as edaravone cannot be administered to patients with renal dysfunction [5]. Edaravone was initially administered for 14 consecutive days, which was followed by a 2-week drug-free period, and was subsequently administered in 28-day cycles. In each cycle, edaravone was administered for 10 days within a 2-week period, according to the standard protocol. Note that edaravone is intravenously administered and is orally unavailable. Therefore, it was administered by a nurse each time and under these conditions, medication compliance was guaranteed.

Data on clinical characteristics, including disease severity based on the Japanese ALS severity classification [23], were retrospectively collected to compare the edaravone and control groups. Disease severity was determined based on the following grading system: grade 1, ability to work or perform housework; grade 2, ability to live independently but inability to work; grade 3, requiring assistance for eating, excretion, or ambulation; grade 4, presence of respiratory insufficiency, difficulty in expectoration, or dysphagia; grade 5, using a tracheotomy tube, tube feeding, or tracheotomy positive-pressure ventilation. The primary study endpoint was TPPV or death, and the follow-up period ended in December 2020.

### 4.2. Statistical Analysis

Differences in clinical characteristics between the two groups were analyzed using the chi-squared test for categorical variables and Student’s *t* test for continuous variables following the verification of normality using the Shapiro–Wilk test. Survival from the disease onset to primary endpoint was analyzed using Kaplan–Meier curves and the log-rank test. The Cox proportional hazard model was used to assess the simultaneous effects of several independent variables on survival. For all analyses, a *p*-value of <0.05 was considered statistically significant. All statistical analyses were performed using SPSS version 25 (IBM, NY, USA).

## 5. Conclusions

The findings of this retrospective single-center study including 45 patients with ALS suggest that edaravone improves survival and maintains its effect over several years without serious adverse events.

## Figures and Tables

**Table 1:** Demographic and baseline characteristics of patients with amyotrophic lateral sclerosis treated with and without edaravone.

	Edaravone Group (*n* = 22)	Control Group (*n* = 23)	*p*
Sex			0.848
Male	14 (64%)	14 (61%)	
Female	8 (36%)	9 (39%)	
Age at onset, years	64.8 ± 11.0	71.4 ± 12.5	0.065
Duration from onset to first visit (months)	9.9 ± 8.6	10.9 ± 8.3	0.705
BMI at first visit	21.8 ± 3.1	21.0 ± 3.5	0.456
ALS severity, average grade	2.0 ± 0.7	2.4 ± 0.8	0.069
Initial symptom			0.485
Limb onset	16 (73%)	18 (78%)	
Bulbar onset	4 (18%)	5 (22%)	
Flail-arm onset	2 (9%)	-	
Treatment with riluzole	18 (81.8%)	14 (60.9%)	0.189
Treatment with PEG	10 (45.5%)	6 (26.1%)	0.175
Treatment with NPPV	2 (9.1%)	1 (4.3%)	0.608
Average duration of edaravone use (months)	26.6 (2–64)		
Average duration from first visit to edaravone treatment (months)	7.2 (1–29)		
Adverse events	Eczema (1; 4.5%)		

ALS, amyotrophic lateral sclerosis; BMI, body mass index; PEG, percutaneous endoscopic gastrostomy; NPPV, noninvasive positive-pressure ventilation.

**Table 2:** Treatment with edaravone and subsequent hazard of death in patients with ALS: Multivariate analysis (*n* = 45).

Variables	Measurement Estimates		Hazard Ratio (95% CI)
	*Coefficient*	*p*	
Treatment with edaravone	−1.011 ± 0.507	0.046	0.36 (0.14–0.98)
Age at onset	−0.009 ± 0.022	0.672	0.99 (0.95–1.03)
Treatment with riluzole	−0.117 ± 0.483	0.809	0.89 (0.35–2.29)
Disease severity grade			
Grade 2/Grade 1	1.160 ± 0.817	0.156	3.19 (0.64–15.8)
Grade 3/Grade 1	1.399 ± 0.936	0.135	4.04 (0.65–25.4)

## Data Availability

All data have been present in main text.
